# Beyond 2D and CNNs: Future Directions for Deep Learning in Periodontal Diagnostics

**DOI:** 10.1016/j.identj.2026.109672

**Published:** 2026-06-11

**Authors:** Kaijie Meng, Shouyin Lv, Jingping Liu

**Affiliations:** Department of Pediatric Dentistry, Hohhot Stomatological Hospital (Inner Mongolia Autonomous Region Stomatological Hospital), Hohhot, Inner Mongolia, China; Digital Center of Oral Implantology, Inner Mongolia People’s Hospital, Hohhot, Inner Mongolia, China; Department of Pediatric Dentistry, Hohhot Stomatological Hospital (Inner Mongolia Autonomous Region Stomatological Hospital), Hohhot, Inner Mongolia, China

Dear Editor—The study by Valente et al^1^ proposed an innovative deep learning model using the YOLOv8I large architecture to detect and classify periodontal osseous defects on periapical radiographs. This approach tackles a significant clinical challenge regarding surgical planning in regenerative periodontology. The attempt to categorise specific defect morphologies into 4 distinct classes rather than merely predicting general bone loss is highly commendable and marks a significant advance. However, we would like to share a few thoughts regarding the methodology and potential future directions.

First, the authors noted that 1 wall defect had the lowest recall, attributing this to the class being the least represented in the training data. While the authors recognised this limitation, they did not employ specific measures to counter the dataset imbalance. Incorporating advanced class balancing techniques may resolve this issue. For example, implementing focal loss during training or using generative adversarial networks for synthetic data generation has proven highly effective. Recent review emphasises that generative adversarial networks are instrumental in overcoming data scarcity and class imbalance in periodontal artificial intelligence applications.^2^

Second, the study used YOLOv8l, which is a state-of-the-art convolutional neural network (CNN). CNNs are proficient at extracting local features. However, detecting ill-defined periodontal defects often requires a broader understanding of the surrounding anatomic context. Vision Transformers leverage self-attention mechanisms to capture global context and long-range spatial relationships.^3^ A recent systematic review highlighted that Vision Transformer models tend to exhibit higher performance than traditional CNNs in various dental image analysis scenarios.^4^ Exploring a Vision Transformer or a hybrid Detection Transformer architecture might further enhance localisation and classification accuracy.

Lastly, the authors correctly acknowledged that 2-dimensional periapical radiographs cannot reliably capture 3-dimensional buccolingual morphology. They stated that confirming the exact number of remaining bony walls typically requires direct intraoperative assessment. To address the intrinsic limitations of 2-dimensional imaging without increasing patient radiation exposure through cone beam computed tomography, a multimodal deep learning approach could be advantageous. Integrating radiographic images with routine clinical tabular data, such as periodontal probing depths and attachment loss, has shown immense promise. A recent study demonstrates that multimodal artificial intelligence models effectively process and reason across diverse data types, capturing complementary information to significantly improve diagnostic accuracy in dentistry.^5^

We hope the above suggestions prove constructive for the research team. Optimising these algorithmic approaches will ultimately facilitate the development of highly reliable diagnostic tools and benefit the broad population of periodontal patients.

## Conflict of interest

None disclosed.
